# Effects of taVNS on physiological responses and cognitive performance during a mental stressor

**DOI:** 10.3758/s13415-025-01341-w

**Published:** 2025-09-04

**Authors:** Lisa Drost, André Schulz, Auriane Möck, Claus Vögele

**Affiliations:** Department of Behavioral and Cognitive Sciences, University of Luxembourg, Belval, 2, avenue de l’Universite, L- 4365 Esch sur-Alzette, Luxembourg

**Keywords:** Brain stimulation, Vagus nerve, PASAT, Working memory, Autonomic nervous system

## Abstract

Transcutaneous auricular vagus nerve stimulation (taVNS) affects autonomic function and enhances cognitive performance by increasing vagal activation and central noradrenergic activity. Nevertheless, the impact of taVNS on acute mental stress remains largely unexplored. This study examined whether taVNS can mitigate the acute sympathetic stress response and improve cognitive performance during a socially evaluated version of the Paced Auditory Serial Addition Task (PASAT). The PASAT is a demanding task that assesses working memory and divided attention and serves as a potent stressor. Forty-one young healthy volunteers were randomly assigned to receive either taVNS stimulation (*n* = 21) at the left cymba conchae or a sham stimulation (*n* = 20) at the ear lobe. Participants received 15-min stimulation before they were challenged with the PASAT while the stimulation continued. Electrocardiogram, electrodermal activity and self-reports of stress and anxiety were collected. Transcutaneous auricular vagus nerve stimulation increased heart rate variability and sympathetic electrodermal activity during the stimulation. Self-reports, cognitive performance and physiological stress responses remained unaffected by taVNS. Physiological measures were highly intercorrelated in participants receiving taVNS. Transcutaneous auricular vagus nerve stimulation did not influence physiological, psychological or behavioral responses to an acute mental/social stressor. The strong intercorrelation between sympathetic and parasympathetic indexes in the taVNS group, however, suggests that taVNS improves autonomic regulation in healthy participants.

## Introduction

Stress is a physiological and psychological response to perceived challenges or threats, which can be adaptive in acute situations by enhancing performance and promoting survival (de Kloet et al., 2005). However, when stress becomes chronic or uncontrollable, it can contribute to a wide range of adverse health outcomes. It has for example been linked to impaired working memory (Gandy et al., 2023) and an increased risk of cardiovascular disease, anxiety, and depression (Hammen, 2005; McEwen, 2017). These conditions are frequently characterized by an imbalance of the autonomic nervous system, with increased sympathetic and reduced parasympathetic activity (Gorman & Sloan, 2000; Thayer et al., 2010). For example, meta-analyses have indicated that patients with major depressive disorder (Kemp et al., 2010) and generalized anxiety disorder (Chalmers et al., 2014) consistently show reduced heart rate variability (HRV), suggesting diminished parasympathetic regulation of heart rate. Hence, there is a growing interest in methods that can mitigate the sympathetic stress response and restore autonomic balance.

Vagus nerve stimulation (VNS) is a promising method to increase vagal (parasympathetic) tone and has shown beneficial effects in conditions with autonomic dysregulation, such as depression (Fang et al., 2016; Rong et al., 2016), tinnitus (Yakunina et al., 2018), and migraines (Song et al., 2023). Non-invasive VNS delivers electrical impulses to the vagus nerve at its auricular or cervical branches. The vagus nerve, being the most important nerve of the parasympathetic nervous system (PNS), contains both ascending afferent and descending efferent fibers (Bonaz et al., 2018). The afferent fibers connect to the nucleus tractus solitarius (NTS) and project from there to neural structures involved in the central autonomic regulation such as the dorsal raphe, the locus coeruleus (LC), the hippocampus and the hypothalamus (Frangos et al., 2015; George et al., 2000). The efferent fibers innervate multiple organ systems involved in the parasympathetic autonomic regulation, including the cardiovascular, respiratory and gastrointestinal systems (Henry, 2002).

Because of its exclusive innervation by the auricular branch of the vagus nerve, the cymba conchae on the outer ear is commonly chosen for taVNS (Ellrich, 2019; Peuker & Filler, 2002). Imaging studies could show that transcutaneous auricular vagus nerve stimulation (taVNS) modulates vagal afferent activity (Badran et al., 2018a, b; Yakunina et al., 2017). Accordingly, acute taVNS modulates HRV (Clancy et al., 2014; Forte et al., 2022; Geng et al., 2022), a peripheral marker of PNS activity, although the evidence remains inconsistent (Wolf et al., 2021). This inconsistency may be due to substantial variability of stimulation parameters across studies, including differences in frequency, stimulus width, stimulation site and duration of the stimulation. Furthermore, it has been suggested that the effects of taVNS may be more pronounced in individuals with reduced baseline HRV (Wolf et al., 2021). In healthy volunteers, taVNS reduces sympathetic stress markers (Badran et al., 2018a, b; Clancy et al., 2014; Jensen et al., 2022) indicating that taVNS shifts the autonomic balance towards a parasympathetic dominance.

There is evidence that taVNS attenuates the acute stress response as measured with different indices of sympathetic activation. Austelle et al. (2024) observed that taVNS mitigated the initial acceleration of heart rate (HR) in response to a (cold) pain stressor compared to sham stimulation. Similarly, Sanchez-Perez et al. (2023) found that taVNS, relative to sham, reduces sympathetic stress reactivity, as indicated with left ventricular-ejection time (LVET) and pre-ejection period to LVET ratio in response to cold and arithmetic stress. In a study of Cuberos Parades et al. (2025), taVNS (compared with sham) did not affect sympathetic stress activation, but decreased cortisol levels during a mental arithmetic stressor. Lastly, De Smet et al. (2023) investigated whether taVNS affected cognition and autonomic response to a psychosocial stressor. While they could not find any direct stimulation effects on the autonomic response, they observed that reduced HRV during stress predicted greater preservative thinking afterward in the sham conditions. However, this relationship was not observed in the taVNS condition.

The cited studies assessed the autonomic stress reactivity with different indices and employed different stress-induction paradigms. Since different stressors engage the autonomic system in distinct ways, it is essential to determine how taVNS affects sympathetic, parasympathetic as well as psychological responses to various forms of stress. In everyday life, mental stressors, which individuals face in a demanding work environment, as well as social stressors from social conflicts, may be most relevant. Their ecological validity is also supported by the fact that chronic occupational and social stressors represent major risks to mental and physical health (Hollis et al., 2022; Kivimäki & Kawachi, 2015). In the current study we therefore investigated the question whether taVNS attenuates psychobiological responses to a psychosocial stressor. For this purpose, we chose a social-evaluated version of the Paced Auditory Serial Addition Task (PASAT; Gronwall, 1977), a well-established arithmetic test that challenges working memory and sustained attention and serves as a potent stressor. In this task, participants hear a series of single-digit numbers and are required to add each new number to the one that immediately preceded it, responding verbally before the next number is presented. Following existing recommendations (Trotman et al., 2019), participants were filmed and asked to perform the task while looking into a mirror to enhance social-evaluative threat and therefore perceived and physiological stress. Moreover, using the PASAT allowed us to calculate a measure of cognitive performance.

Previous research has shown that taVNS has beneficial effects on several aspects of cognition, including decision-making (Kühnel et al., 2020), response inhibition (Beste et al., 2016; Jongkees et al., 2018), attention (Ventura-Bort et al., 2018), and working memory (Sun et al., 2021; Tan et al., 2024), with stronger effects for high cognitive load. For instance, Tan et al. (2024) reported improved performance in a 4-back task following vibrotactile VNS and Sun et al. (2021) observed that taVNS enhances performance in a 3-back spatial task, but not in the 1-back task. While the overall pattern is promising, some inconsistencies remain in the literature, and not all findings have been successfully replicated (for review, see Ridgewell et al., 2021).

This effect is thought to be driven by taVNS increasing the activation of brain regions that are involved in cognitive processes, such as the PFC (Badran et al., 2018a, b). Moreover, taVNS is suggested to increase the release of norepinephrine (NE) from the LC and the release of GABA from the NTS (Colzato & Beste, 2020). This assumptions is based on the anatomical pathway of the auricular branch of the vagus nerve which innervates the NTS and subsequently relays to the LC (Frangos et al., 2015; George et al., 2000). Imaging studies have shown that taVNS enhances LC activity (Yakunina et al., 2017). Moreover, taVNS has been found to modulate the P3 amplitude in healthy individuals (Gurtubay et al., 2023; Ventura-Bort et al., 2018). This ERP component is closely linked to attention and working-memory processes and is influenced by phasic NE release from the LC (Nieuwenhuis et al., 2005). However, it remains unclear whether taVNS can also improve cognitive performance under acute stress.

To address this gap, this study examined the effects of active taVNS on the stress reactivity to the PASAT, using biomarkers of as SNS and PNS activity. HR, electrodermal activity (EDA), and T-wave amplitude (TWA) were used as indices of SNS activation. Specifically, increases in HR and EDA, as well as decreases in TWA, are commonly associated with heightened sympathetic arousal. In contrast, parasympathetic nervous system (PNS) activity was indexed using HRV, with higher HRV reflecting greater parasympathetic tone. We hypothesize that 1) active taVNS would increase vagal tone (as reflected by HRV) and mitigate the sympathetic stress response (HR, EDA, TWA) compared with sham stimulation. We also expected that 2) participants in the taVNS condition would perform better in the PASAT task, as reflected by higher accuracy scores.

## Methods

### Participants

Forty-one volunteers took part in single experimental sessions (age: 19–28, mean = 22.5 years, *sd* = 2.51, women = 23 participants). Participants were recruited via the university study registration website and by distributing print-flyers on the campus. They were eligible if they had no current mental or physical illness, if they were free of regular medication intake (except for occasional non-steroidal pain killers), and non-smokers. Participants were asked to abstain from heavy exercise, alcohol, and caffeine consumption the night before as well as on the morning of the experimental session. The study was conducted in accordance with the ethics regulations of the university of Luxembourg. Written informed consent was obtained from all participants, and they received a 10€-gift voucher as a reimbursement for their participation in the study. Participants were randomly allocated to one of two groups: real taVNS (*n* = 21) or sham taVNS (*n*=20). Groups did not differ in their age or BMI (*t*s < 1; see Table 1).

**Table 1:** Descriptive statistics by group

Group	Age	BMI	Stimulation intensity (mA)	*N* men	*N* women
Sham	22.6 ± 0.6	22.0 ± 0.7	2.1 ± 0.1	10	10
taVNS	22.5 ± 0.6	21.5 ± 0.5	1.7 ± 0.1	8	13

mean ± *se*

### Experimental protocol

**Fig. 1:**
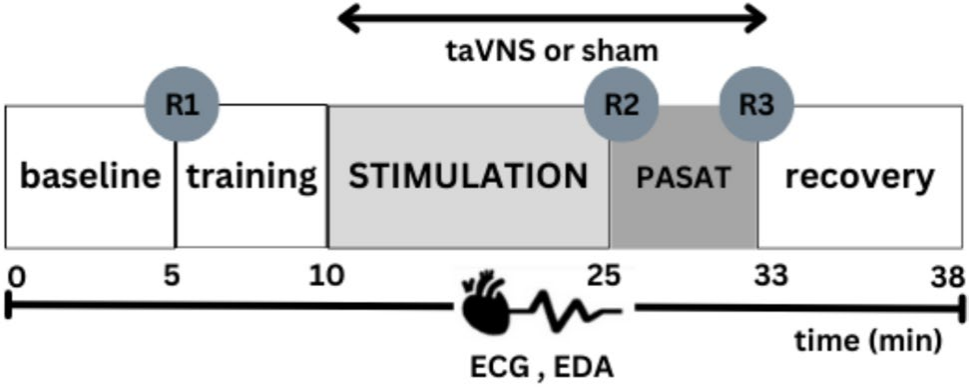
Experimental procedure. R1–R3: Self-reports of stress and anxiety

Figure 1 provides an overview of the general experimental procedure. After arrival at the lab, participants were informed about the experimental procedure, signed the informed consent form and filled out questionnaires assessing demographic information. After ECG and EDA electrodes were attached, a 5-min baseline measurement started. Then, participants underwent a 5-min training for the PASAT before receiving active taVNS or sham stimulation for 15-min. Next, they were challenged with the PASAT while the stimulation continued. The participants provided self-reports of perceived stress and anxiety at three time-points: before the PASAT training, after the stimulation, and after the PASAT. After the PASAT, a 5-min resting-state measurement was conducted and then electrodes were detached. Experimental timing was controlled with PsychoPy (Peirce et al., 2019; Version 2023.2.3). Finally, participants were debriefed and received their reimbursement.

### Transcutaneous auricular vagus nerve stimulation

For taVNS, the NEMOS device (Cerbomed GmbH, Erlangen, Germany) was attached to the left cymba conchae for the real stimulation, since this area is exclusively innervated by the auricular branch of the vagus nerve (Ellrich, 2011). For sham stimulation, we attached the electrodes to the left earlobe since this area is not innervated by the vagus nerve (Ellrich, 2011). We have exclusively chosen the left ear to avoid possible cardiac side effects, such as bradycardia (Kim et al., 2022). Participants were stimulated with predefined settings of the device consisting of a frequency of 25 Hz and a pulse width of 200–300 µs, with an interval of 30-s on/30-s off. The amplitude was calibrated individually for each participant so that they would feel a strong sensation that was not perceived as painful, following a procedure similar to that described by Fischer et al. (2018). Initially, the device was set to a stimulation intensity of 1.5 mA. Participants first experienced a 30-sec stimulation cycle to become accustomed to the sensation. Thereafter, they verbally rated their discomfort on a scale from 1 (*no discomfort*) to 10 (*extremely uncomfortable*). The intensity was then increased to 2.0 mA and subsequently adjusted in 0.1 mA increments. At each step, participants were asked whether they experienced any discomfort. Once a participant reported a painful sensation, the intensity was reduced to the previous, non-painful level and used as the final stimulation setting. Stimulation intensity was higher in the sham group, *t*(38.9) = 2.02, *p* =.049 (see Table 1).

### Paced Auditory Serial Addition Task

The mental arithmetic PASAT (Gronwall, 1977) was used as mental stressor. A series of single-digit numbers was presented over speakers and participants were requested to add the last two numbers they heard and say the answer out loud. A total of 225 digits were presented. As suggested by Trotman et al. (2019), we gradually shortened the interstimulus interval (ISI) for the digit presentation from 2.0 s to 1.6 s, and finally to 1.2 s. To further increase perceived stress, a social evaluation component was added. Before the start of the PASAT we set up a camera and informed participants that they would be videotaped and their performance judged by an external evaluator. Additionally, a mirror was placed in front of the participants, and they were instructed to watch themselves while performing the task. Verbal responses were recorded with a microphone. The entire procedure lasted approximately 8 min. Performance in the PASAT was quantified in terms of percentage of correct responses. One person (sham) did not reply at all and was excluded from analysis.

### Physiological data processing

ECG and EDA signals were recorded continuously with a sampling rate of 2000 Hz and amplified with a Biopac MRI Smart Amplifier (BIOPAC Systems, Inc). The ECG signal was measured using three Ag/AgCl gel electrodes according to the Einthoven lead II configuration. EDA was recorded by placing two electrodes on the middle phalanges of the index finger and middle finger of the left hand. For analysis, only the first 5 min of each period (i.e., baseline, stimulation, stress, and recovery) were included. ECG and EDA data were processed using NeuroKit2 (Makowski et al., 2021) in Python 3.10.13 within Spyder 6.0.0.

ECG data were filtered using a high-pass (0.05 Hz) and a 50 Hz notch filter. R-waves in the ECG were automatically detected and artifacts in the RR time series were corrected using the internal algorithm of the Kubios method. HR and TWA were averaged for each participant over the periods of interest (i.e., baseline, stimulation, stress, and recovery). We used the root mean square of successive differences (RMSSD) as index of HRV, as this measure is sensitive to parasympathetic activity and was demonstrated to be a robust indicator in short time segments. RMSSD was calculated for each participant and period of interest.

Continuous EDA data were low-pass filtered at 3 Hz. EDA power was calculated within the frequency band of 0.045–0.25 Hz as a sensitive marker of sympathetic activity (Posada-Quintero et al., 2016) and averaged for each participant and period of interest.

### Self-reports

Participants provided ratings of perceived stress and anxiety on a visual-analog scale (VAS), ranging from 1 (*not at all*) to 5 (*very much*).

### Data reduction and statistical analysis

All physiological data were tested for baseline differences using *t*-tests for independent samples, and delta values were calculated by subtracting the mean of the baseline period from each subsequent period (i.e., stimulation, stress, and recovery) for each participant and parameter. Outliers were defined as values more than ±3 standard deviations from the mean. Due to the presence of outliers across multiple periods, the following exclusions were made: one participant from the sham group was excluded from the HR analysis, one participant from the taVNS group was excluded from the TWA analysis, one participants from the sham group and one from the taVNS group were excluded from the EDA analysis. Additionally, three outlier values were excluded from the RMSSD analysis, and one outlier value was excluded from the EDA analysis.

Linear mixed models were fitted to examine the effect of period and group on all outcome variables while controlling for baseline values. Each model included fixed effects for group, period, the Group × Period interaction, the respective baseline value of the outcome variable as well as random intercepts for participants to account for repeated measures. Degrees of freedom were adjusted using the Satterthwaite method and effect sizes (*η*^*2*^*ₚ*) are reported for significant fixed effects. In case of significant results, contrasts were calculated and Bonferroni correction applied to correct for multiple testing. Furthermore, Pearson correlations between all reactivity parameters and cognitive performance were calculated for each group. To test the equality of the correlation matrices Jennrich test was used (Jennrich, 1970).

All analysis were performed in R Studio (Version 4.2.3; RStudio Team, 2020) using the packages *lme4* (Bates et al., 2015), *effectsize* (Ben-Shachar et al., 2020), and *emmeans* (Lenth, 2025). In figures and tables, the means and the standard errors of mean (*se*) are presented.

## Results

### Physiological parameters

**Fig. 2:**
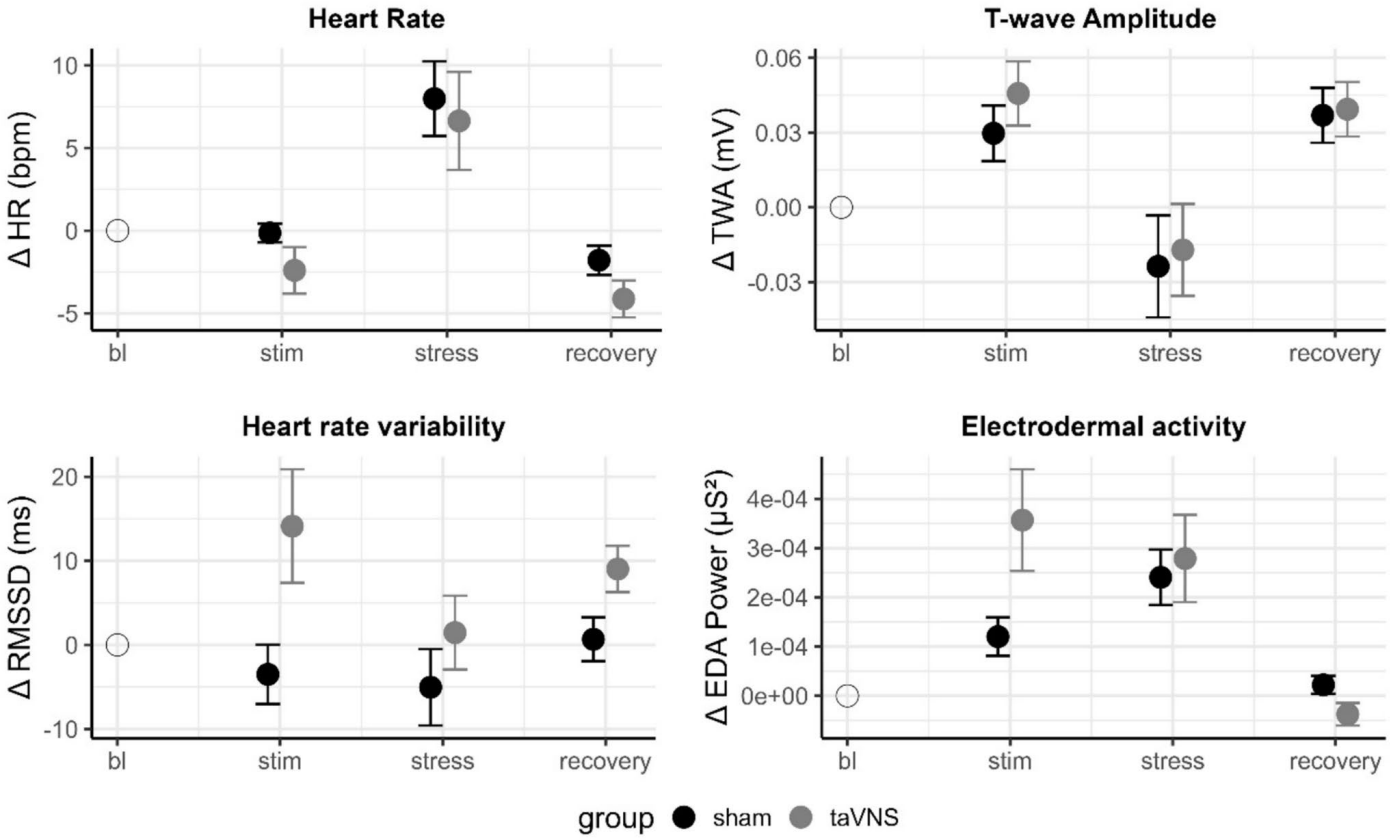
Mean changes (*se*) of physiological signals from baseline (bl) during stimulation (stim), stress and recovery phase

Figure 2 illustrates the mean changes of physiological parameters across periods.

#### Heart rate (HR) 

The main effect of period, *F*(3,14) = 23.22, *p* <.001, *η*^*2*^*ₚ* = 0.38, indicated significant higher HR during stress compared to stimulation, *t*(114) = 6.46, *p* <.001, and recovery, *t*(114) = −7.73, *p* <.001. Lower baseline HR was associated with increased HR responses, *F*(1,37) = 6.28, *p* =.017,* η*^*2*^*ₚ* = 0.15; taVNS had no significant effect on HR responses, *F*(1,37) < 1, or on the time course of HR responses, *F*(3,114) < 1.

#### T-wave amplitude (TWA)

The main effect of period, *F*(3,114) = 15.87,* p* ≤.001, *η*^*2*^*ₚ=* 0.29, indicated higher TWA during stimulation compared to baseline, *t*(114) = 3.66, *p* =.001, and significantly lower TWA during stress compared to the stimulation period, *t*(114) = −5.64, *p* <.001, and the recovery period, *t*(114) = 5.69, *p* <.001. Again, we did not observe any stimulation effects on TWA responses, *F*(1,37) < 1, or the time course of TWA responses, *F*(3,114) < 1. Baseline TWA values had no effect, *F*(1,37) < 1.

#### RMSSD 

The taVNS group showed overall higher RMSSD compared to the sham group, *F*(1,37.8) = 4.27, *p* =.046, *η*^*2*^*ₚ=* 0.10. The stressor had an effect on RMSSD, *F*(3,114.3) = 3.28, *p* =.024, *η*^*2*^*ₚ*= 0.08, with significantly lower RMSSD during stress compared with the stimulation period, *t*(114.45) = −2.55, *p* =.040, and recovery, *t*(114.5) = 2.50, *p* =.042. Moreover, taVNS affected the time course of the RMSSD response, *F*(3,114.3) = 3.04, *p* =.032, *η*^*2*^*ₚ* = 0.07, with significantly higher RMSSD in the taVNS than in the sham group during stimulation, *t*(109.7) = −3.31, *p* =.001, but no difference after the stressor. Lower baseline RMSSD was associated with higher RMSSD across time and groups, *F*(1,38.78) = 4.54, *p* =.049, *η*^*2*^*ₚ=* 0.10.

#### **EDA**

EDA power increased in response to the stressor, *F*(3,105.6) = 17.46, *p* <.001, *η*^*2*^*ₚ=* 0.33, with higher EDA power during stimulation, compared with baseline, *t*(105.4) = 4.95, *p* <.001, and a lower EDA power in the recovery period compared with stress, *t*(105.8) = −5.26, *p* <.001. The stimulation had an effect on the course of the EDA power over time, *F*(3, 105.6) = 3.17, *p* =.027, *η*^*2*^*ₚ=* 0.08, with higher EDA power responses in the taVNS group than in the sham group during the stimulation period, *t*(104.9) = −2.58, *p* =.011, but no difference after the stressor. We observed no main effect of stimulation, *F*(1,37.78) < 1, nor an effect of baseline EDA power, *F*(1,31.7) =1.80, *p* =.189.

### PASAT performance

Groups did not differ in their PASAT performance, *t*(39) = 0.35, *p* =.727.

### Self-reports

Mean values of stress and anxiety are presented in Table 2. Participants receiving taVNS stimulation reported higher overall stress, *F*(1,115)= 4.42, *p* =.038, *η*^*2*^*ₚ=*.04, and anxiety levels, *F*(1,38) = 6.72*, p* =.013, *η*^*2*^*ₚ =*.15, compared with sham. Stress, *F*(2,116) = 83.66, *p* <.001, *η*^*2*^*ₚ =*.59, and anxiety ratings, *F*(2,78) = 31.46, *p* <.001, *η*^*2*^*ₚ =.*45, changed over time, with higher self-reported stress after the stimulation, *t*(78) = 2.38, *p* =.040, and after stress, *t*(78) = 9.82, *p* <.001. For anxiety, participants reported higher anxiety after stress, *t*(78) = 6.03, *p* <.001, but not after the stimulation, *t*(78) = 1.45, *p* =.303. Higher baseline stress ratings were associated with lower overall stress ratings, *F*(1,116) = 24.63, *p* <.001, *η*^*2*^*ₚ =*.18. Similarly, higher baseline anxiety was associated with lower anxiety rating during the experiment, *F*(1,38) = 22.23, *p* <.001, *η*^*2*^*ₚ =*.37. The different stimulation protocols (taVNS vs. sham) did not affect changes of self-reported stress, *F*(2,116) < 1, or anxiety ratings, *F*(2,78) = 1.48,* p* =.233, over time.

**Table 2:** Self-reported stress and anxiety; visual analog scale ranging from 1 (not at all) to 5 (*very much*)

Group	Stress			Anxiety		
	R1	R2	R3	R1	R2	R3
Sham	1.6 ± 0.2	1.8 ± 0.1	3.2 ± 0.2	1.6 ± 0.2	1.5 ± 0.2	2.5 ± 0.3
taVNS	1.8 ± 0.2	2.2 ± 0.2	3.6 ± 0.2	1.8 ± 0.2	2.3 ± 0.2	3.1 ± 0.2

mean ± *se*, R1= pre-stimulation, R2= post-stimulation, R3 = post-stress. For illustration purposes only, the raw data are displayed in this table

### Correlation analysis

Tables 3 and 4 present the correlation matrices for the taVNS and sham group, respectively. In the taVNS group, significant correlations were observed between most physiological variables across both the stimulation and stress phases. In contrast, the sham group exhibited significantly fewer significant correlations. The correlation matrices significantly differed between both groups, χ^2^(28) = 116.4, *p* <.001.

**Table 3:** Pearson correlations between the baseline-corrected physiological data during stimulation and stress in the taVNS group

	HR stim	HR stress	TWA stim	TWA stress	RMSSD stim	RMSSD stress	EDA stim	EDA stress
HR stim	1	.83***	−.72***	−.66**	−.50*	−.45*	−.20	.13
HR stress		1	−.59**	−.69***	−.50*	−.47*	−.44	−.41
TWA stim			1	.70***	.46*	.57**	.10	−.01
TWA stress				1	.48*	.50*	.164	.12
RMSSD stim					1	.89***	.51*	.59**
RMSSD stress						1	.34	.46*
EDA stim							1	.75***
EDA stress								1

*HR*, heart rate; *TWA,* T-wave amplitude; *RMSSD,* root mean square of successive differences; *EDA,* Sympathetic Index of electrodermal activity. Physiological data assessed during stimulation (stim) or stress (stress)

*p <.05; **p <.01; ***p <.001

**Table 4:** Pearson correlations between the baseline-corrected physiological data during stimulation and stress in the sham group

	HR stim	HR stress	TWA stim	TWA stress	RMSSD stim	RMSSD stress	EDA stim	EDA stress
HR stim	1	.46*	−.10	−.22	−.40	−.30	.02	.14
HR stress		1	−.10	−.69**	−.25	−.63**	−.14	−.20
TWA stim			1	.44	.44	.19	.01	.23
TWA stress				1	.52*	.74***	.14	.49
RMSSD stim					1	.82***	.20	.29
RMSSD stress						1	.15	.30
EDA stim							1	.70***
EDA stress								1

*HR,* heart rate; *TWA*, T-wave amplitude; *RMSSD,* root mean square of successive differences; *EDA,* Sympathetic Index of electrodermal activity. Physiological data assessed during stimulation (stim) or stress (stress)

*p <.05; **p <.01; ***p <.001

## Discussion

The aim of this study was to investigate the effects of acute taVNS on autonomic responses and cognitive performance under mental stress. Our findings show that taVNS increases RMSSD and EDA power during stimulation, indicating successful modulation of autonomic processes. In contrast, the stimulation did not modulate physiological or psychological responses to the acute stressor. Furthermore, we observed significant correlations between autonomic markers during stimulation and stress in the taVNS group.

HR increased during stress, regardless of taVNS or sham stimulation, consistent with findings of De Smet et al. (2023), using the TSST, and Cuberos Paredes et al. (2025), employing a mental arithmetic stressor. However, it contradicts recent findings by Austelle et al. (2024), who reported that taVNS attenuated the initial acceleration of HR in response to cold stress compared with sham. Their observed effect was, however, transient, lasting only 40 s. This suggests that taVNS-induced changes in HR may be short-lived. Another explanation for these discrepancies would be that the type of stressor Austelle et al. (2024) applied—ischemic cold stress that induces pain—may elicit specific physiological responses that might be modulated by taVNS. It could be argued, for example, that taVNS specifically affects the central autonomic network (Thayer & Lane, 2000), which is important for the down-regulation of affective responses to pain stimuli, thereby specifically affecting stress reactivity to pain stimuli. In contrast, we, De Smet et al. (2023), and Cuberos Paredes et al. (2025) applied a psychosocial and/or mental stressors, which may have higher ecological validity, as these stress components are more representative for stressors encountered in everyday life. One may speculate, therefore, that taVNS is ineffective in regulating stress-induced reactivity to stressors involving these characteristics. Furthermore, Badran et al., (2018a, b) investigated how different stimulation protocols affect HR. Their results suggest that the effects of taVNS on HR depend on frequency and pulse width of stimulation, with the combination of 500 µS and 10 Hz producing the greatest impact. This implies that different stimulation parameters than those used in our study might be more effective in eliciting HR responses. Yet there is still no consensus on the optimal stimulation protocols (Butt et al., 2020; D’Agostini et al., 2023; Shao et al., 2023).

Our findings on TWA are in line with previous studies showing decreased or inversed T-wave following mental stress (Rau, 1991; van Lien et al., 2015). Given that TWA reflects cardiac repolarization and is considered a specific biomarker of sympathetic activity (Furedy, 1987), the absence of a taVNS group effect suggests that taVNS does not modulate cardiac repolarization. This aligns with the finding that other cardiac indices, such as the pre-ejection period (PEP), may be more sensitive markers of sympathetic activation (Drost et al., 2022). Accordingly, Gurel et al. (2020) and Sanchez-Perez et al. (2023) found that taVNS reduces sympathetic cardiac reactivity measured with PEP, left-ventricular ejection time, and vasoconstriction. Future studies are needed to investigate the suitability of other cardiovascular indicators for reflecting autonomic activity modulated by taVNS.

HRV indices for vagal control over heart rate are amongst the most frequently studied biomarkers in taVNS research. This focus is plausible given the vagus nerve’s anatomical connection to the sinoatrial node, allowing it to influence heart rate and HRV (Standish et al., 1994). In our study, we observed a robust increase in RMSSD during stimulation only. Several studies have reported increases in HRV during taVNS, reflected in the parameters RMSSD, high-frequency (HF) power and the low-frequency/high-frequency (LF/HF) ratio (Forte et al., 2022; Geng et al., 2022; Keute et al., 2021). However, other studies reported effects on specific parameters such as SDNN (De Couck et al., 2017), HF-HRV (Lamb et al., 2017) or LF-HF ratio (Clancy et al., 2014) without corresponding changes in other parameters. Notably, studies differ in stimulation duration, stimulation parameters (e.g., frequency, pulse width), sham protocols, and the choice of HRV indices. While RMSSD and HF-HRV are widely considered reliable indicators of vagal activity, the LF/HF ratio is influenced by both sympathetic and parasympathetic inputs (Shaffer & Ginsberg, 2017). Furthermore, the recording duration might also play a critical role. For example, studies reporting increased RMSSD often used short stimulation periods (≤10 min) in young, healthy participants (Forte et al., 2022; Geng et al., 2022). Similarly, we limited our HRV analysis to the first 5 min of stimulation. These findings suggest that taVNS effects on HRV may be most prominent shortly after stimulation onset. Moreover, in our study the baseline HR and RMSSD were significantly associated with cardiac responses. This is consistent with previous evidence suggesting a relationship between baseline HRV and the HRV response to taVNS (Clancy et al., 2014).

Given that taVNS is assumed to increase vagal tone, we hypothesized that it decreases sympathetic activity as indexed by EDA power. However, previous studies found no effect of taVNS on EDA (De Smet et al., 2023; Höper et al., 2022; Sanchez-Perez et al., 2023). Contrary to these findings and our expectations, we observed an increase in sympathetic EDA power during taVNS. The differing results across the studies may be due to the use of different indices to quantify EDA, such as integrated skin conductance response (Höper et al., 2022), tonic EDA (De Smet et al., 2023; Sanchez-Perez et al., 2023) and the sympathetic index of EDA. Notably, our results align with the assumption that taVNS increases noradrenergic activity in the LC (Ludwig et al., 2021), potentially leading to heightened sympathetic arousal. In support of this notion, prior research has demonstrated that taVNS increases pupil dilation (Ludwig et al., 2024; Skora et al., 2024), another sympathetic marker associated with arousal (Aston-Jones & Cohen, 2005; Ebitz & Platt, 2015). One may speculate, therefore, that the physiological response pattern related to taVNS is more complex than an overall increase of vagal activation, with a differential stimulation of parasympathetic and sympathetic pathways instead, as reflected by RMSSD and EDA.

In our study, taVNS did not affect cognitive performance, contradicting findings from Zhao et al. (2023), who reported improved working memory following taVNS under sleep-deprivation stress. Nevertheless, sleep deprivation likely imposes greater systemic strain compared with the PASAT used in our study. Hence, it may be concluded that taVNS might be more effective in enhancing cognitive performance in severe stress conditions. Moreover, Tan et al. (2024) found increased working memory during an n-back task after vibrotactile stimulation of the vagus nerve with a frequency of 6 Hz. We suggest future studies that systematically investigate the differences in stimulation protocols to conclude whether differences in that could account for discrepant findings.

Participants reported higher stress and anxiety following the stress induction, confirming the effectiveness of the stressor on a self-reported level. In contrast to earlier findings, we observed that taVNS increased stress and anxiety. Previous studies have shown that taVNS can reduce self-reported stress (Dos Reis et al., 2024) and anxiety (Ferreira et al., 2024) in healthy individuals. Those effects were, however, observed after repeated stimulation. Moreover, we observed a strong effect of baseline stress and anxiety ratings: Participants with higher baseline scores reported lower overall stress and anxiety across the session. This might indicate that individuals with elevated baseline affect may respond differently to taVNS. Future studies should examine whether baseline affective states moderates taVNS efficacy, ideally using longer-term protocols.

Correlation analyses revealed strong associations among physiological measures in the taVNS group both during stimulation and stress, suggesting enhanced overall autonomic regulation. Specifically, the positive correlation between RMSSD and EDA power in the taVNS group implies a synergistic effect in autonomic regulation, where both branches (parasympathetic and sympathetic) are coordinated rather than opposing each other. Coactivation of the autonomic branches has been previously documented, and evidence suggests that this coordination depends on contextual as well as individual factors (Berntson et al., 1993; Cacioppo et al., 1994; Weissman & Mendes, 2021). In contrast, the sham stimulation did not produce similar autonomic interactions, which suggests that taVNS may facilitate more efficient autonomic regulation and therefore support physiological flexibility and adaptability.

## Limitations

Several limitations should be considered when interpreting these findings of this study. First, we used the PASAT as combined mental/social stressor. Nevertheless, whether our findings generalize to other facets of acute stress remains to be investigated in future studies. Second, the study sample consisted solely of healthy university students. Further investigation is needed to determine whether our results can be generalized to clinical populations or individuals with autonomic dysregulation. Third, several studies have suggested age and sex differences in the effects of taVNS. Therefore, our results should be replicated in more diverse and heterogeneous populations to ensure broader applicability. Fourth, the use of a single stimulation protocol limits the ability to draw conclusions about the optimal parameters for taVNS. Although a 30-s ON/30-s OFF protocol is common in short-term, experimental taVNS studies, this differs from protocols used for long-term stimulation in mental or neurological disorders (e.g., 30 s ON/5 min OFF). Future research should explore the effects of varying stimulation intensities, frequencies, duty cycles and durations to better understand how these factors influence autonomic and cognitive responses. Lastly, although previous research has indicated that taVNS is generally safe (Kim et al., 2022), in our study we only assessed side effects informally through verbal inquiry, asking participants whether they were experiencing any adverse effects. While no such effects were reported, the absence of a structured, written assessment limits the thoroughness of our safety evaluation. Future studies should incorporate systematic and documented monitoring of side effects to more accurately assess safety and tolerability of taVNS.

## Conclusion

In conclusion, our results demonstrate that taVNS induces changes in parasympathetic and sympathetic biomarkers during stimulation. Physiological measures were highly correlated during stimulation as well as during stress. This implies that taVNS might help to coordinate the autonomic regulation in healthy volunteers.

## Data Availability

Data or materials for the experiments are available upon request, and none of the experiments was preregistered.

## Code availability

The code for data analysis is available upon request.
